# COVID-19 Vaccine Early Skepticism, Misinformation and Informational Needs among Essential Workers in the USA

**DOI:** 10.3390/ijerph182413244

**Published:** 2021-12-15

**Authors:** Elena Savoia, Maxwell Su, Rachael Piltch-Loeb, Evelyn Masterson, Marcia A. Testa

**Affiliations:** 1 Department of Biostatistics, Harvard T.H. Chan School of Public Health, Boston, MA 02115, USA; masu@hsph.harvard.edu (M.S.); piltch-loeb@hsph.harvard.edu (R.P.-L.); testa@hsph.harvard.edu (M.A.T.); 2 Emergency Preparedness Research, Evaluation & Practice (EPREP) Program, Harvard T.H. Chan School of Public Health, Boston, MA 02115, USA; emasterson@hsph.harvard.edu

**Keywords:** COVID-19, vaccine acceptance, misinformation

## Abstract

This study presents the results of a survey of 1591 hesitant U.S. essential workers, conducted over Pollfish in December 2020 when they were the only group eligible for the vaccine, aiming to describe their concerns regarding COVID-19 vaccine safety, effectiveness and distribution policies. We computed frequencies using the SAS software for each answer, using chi-squared statistics and Cochran–Armitage trend tests to determine how informational needs differ by age, gender, level of education, race, source of COVID-19 information and levels of vaccine acceptance. The results of this study show that freedom of choice, equal access to the vaccine and being able to live a life with no restrictions once vaccinated were important concerns since the early days of the distribution campaign, with 53% (836/1591), 42% (669/1591) and 35% (559/1591) of hesitant respondents, respectively, indicating they would be more likely to receive the COVID-19 vaccine if they felt these issues were satisfactorily addressed. Early risk communication and immunization campaign strategies should address not only the reported efficacy and safety of new vaccines, but, as equally important, the population’s perceptions and beliefs regarding personal choice, effectiveness and adverse consequences.

## 1. Introduction

An analysis of 39 nationally representative polls, conducted between August 2020 and February 2021, suggests that approximately 11% of the U.S. public is skeptical about receiving the COVID-19 vaccine and that 17–22% are “definitely not” interested in receiving it [1,2]. At the time of this writing, in December 2021, approximately 72% of the U.S. adult population is fully vaccinated [2]. If intent, as expressed in the polls, is followed by behavior, we may be hitting a plateau in vaccination rates among U.S. adults. This situation could leave us with approximately 74 million adults at risk of experiencing symptoms of COVID-19, including people working in services that are important to the functioning of our society such as healthcare, education, transportation and other essential services. Workers in these sectors have been critical to societal functioning before and throughout the pandemic.

In the United States, during the first wave of shelter-in-place and stay-at-home orders, only “essential” workers were allowed to continue working at their regular place of business. Federal, state- and city-level governments issued guidance on which sectors and industries they considered “essential” under pandemic-related restrictions. The Cybersecurity and Infrastructure Security Agency (CISA) of the U.S. Department of Homeland Security defines “essential workers” as those who conduct a range of operations and services that are typically essential to maintain critical infrastructure [3]. “Critical infrastructure” is a large umbrella term encompassing many sectors, from energy to defense to agriculture. Importantly, during the pandemic, essential workers were not only at increased occupational risk for exposure to COVID-19 [4,5] but also at increased risk of suicidal ideation and mental health distress, factors that underscored the work burden they faced during the crisis [6] and how the pandemic impacted their lives beyond the risk of infection.

People working as “essential workers” have been included in the priority groups for vaccination since the early days of the vaccine campaign [7]. Moreover, the U.S. Advisory Committee on Immunization Practices (ACIP) recommended that, apart from the elderly, the first supplies of the COVID-19 vaccine be allocated to healthcare workers, frontline essential workers and other people working in essential and critical infrastructure sectors [8,9]. The CDC conducted a poll of priority groups for the vaccine in September 2020, prior to the rollout of the vaccine, to understand vaccine acceptance among these workers. A second poll was conducted in December 2020 during the first phase of the vaccine distribution campaign. While the second poll showed a substantial increase in vaccine acceptance compared to the previous one, the highest acceptance rate reported was not greater than 60% [10].

The success of the reopening of society depends on the success of the immunization campaign, until the seroprevalence and mucosal immunity of the general population will fully transition SARS-CoV-2 into an endemic state. Besides the obvious need to prevent hospitalizations and deaths, approximately 140,000 lives were estimated to be saved by the vaccine in the US alone [11], it is also important to the successful reopening of society that labor productivity be maintained over time, particularly among those who play an essential role in keeping key services functioning. While the coverage needed to achieve herd immunity is difficult to define and still an object of open debate [12], current coverage rates can be discussed in terms of susceptible to SARS-CoV-2 infection and to the progression to serious clinical complications due to COVID-19. As of mid-March 2021, roughly half (48%) of essential workers reported that they have already received at least one dose of the COVID-19 vaccine or will recieve it as soon as they can. Unfortunately, this is a lower share than amongst individuals who are employed in other professions (69%). Furthermore, essential workers are more likely than those who can work from home to say they will “definitely not” receive the vaccine (21% vs. 7%) and are more likely to say that they will receive it “only if required” (11% vs. 3%) [13].

The vaccine decision-making process of essential workers can have consequences beyond their own health, in particular due to the risk of transmitting the virus to patients susceptible to more severe infections. Essential workers, especially healthcare workers, may play a key role in health and risk communication to other people with whom they interact. Healthcare professionals are often seen as trusted messengers for their communities in conveying general health information and health information specific to the vaccine. Poll respondents frequently report that they trust their doctor and other healthcare professionals to share information regarding vaccines’ safety [1]. There is a lack of data on the specific role played by other essential workers, such as police officers, firefighters, transportation personnel and teachers as trusted messengers. However, members of an individual’s network can be some of the most influential messengers in shifting behavior [14], and it is reasonable to think that these individuals also play a key role as trusted messengers within their networks. Therefore, it is important to understand vaccine acceptance among essential workers for two reasons. Firstly, they are at an increased risk for contracting COVID-19 given their interactions with the public, and, secondly, they may play an important role as trusted messengers to others in their community. Understanding what type of additional information could be provided to them to reduce their skepticism is important for building an effective communication strategy directed towards not only essential workers, but the population that surrounds them.

In this study, we surveyed essential workers in the United States prior to the vaccine rollout, in December 2020, including people working in the healthcare sector, nursing homes, public health, transportation and other essential services and asked what type of information would make them more likely to receive the COVID-19 vaccine. We believe that, even if vaccine demand and supply have changed during the first nine months of the vaccination campaign (December 2020–November 2021), these data are still informative and relevant to the current discourse on vaccine acceptance because, during emergencies, people tend to believe the very first messages and information they receive [15]. As such, what they believed in December 2020 is relevant today because it may still be influencing their actions, including the acceptance of a vaccine booster if already vaccinated. The primary goal of our study was to explore what type of information those skeptical about the vaccine needed, at the time the vaccine was offered to them, to make them more willing to accept it, and explore the association between sociodemographics, use of social media (as the main source of COVID-19 vaccine information), and strength of opinions about getting or not getting the vaccine (vaccine opinion certainty). 

## 2. Materials and Methods

### 2.1. Study Design

We used a cross-sectional online survey study design. The survey was implemented via mobile phones using the survey platform Pollfish and limited to essential workers aged ≥ 18 residing in the USA. Like third-party advertising companies, Pollfish pays mobile application developers to display and promote the surveys to their users using crowdsourcing. The survey was implemented between 13 December 2020 and 23 December 2020. A screening question was used to identify respondents belonging to one of 19 job categories (essential workers) determined to be priority groups for vaccine distribution based on national guidance available at the time of the survey [16,17]. The study protocol and survey instrument were approved by the Harvard T.H. Chan School of Public Health Institutional Review Board on 8 December 2020 (protocol #:IRB20-2032). Consent to participate in the survey was asked to participants prior to start responding to the questions. A copy of the questionnaire is provided as Supplementary Materials to this manuscript. In particular, questions related to misinformation were created based on an analysis of most frequently reported tropes and misinformation narratives performed in a previous study [18]. The questions were tested on a small sample (20 individuals) and the feedback incorporated in a revised version of the questionnaire prior to implementation. 

### 2.2. Statistical Analysis

We first focused our analysis on individuals who expressed acceptance about receiving the COVID-19 vaccine by excluding those that reported being very likely to receive it. Among the group of hesitant individuals, we analyzed responses to the following question “What would be important for you to know to make you more likely to take the COVID-19 vaccine?”. We computed frequencies for each answer—which we will refer to here as “informational needs”. We used the chi-squared test of independence, followed by Cochran–Armitage trend tests, when appropriate, to explore differences in informational needs by age, gender, level of education, race and source of COVID-19 vaccine information (social media vs. traditional media). We also computed the same statistics to study the association between informational needs and levels of vaccine acceptance. When the chi-squared statistic was significant, we explored which categories of the variable were responsible by examining the squared Pearson residuals, which are roughly distributed as a chi-squared with one degree of freedom, with cells having a value greater than 3.84 being noted as having observed counts greater/less than expected. For the binary variables age and source of COVID-19 vaccine information, a significant chi-squared statistic implies the two categories differ in informational needs, so we report the differences between the two categories regardless of the size of the squared Pearson residuals. Descriptive statistics were computed using the statistical package SAS. Subsequently, to further explore the association between vaccine opinions, sociodemographic characteristics and use of social media, we applied logistic regression. In this model we also included respondents very likely to receive the vaccine and created the dependent variable “vaccine certainty” (coded as 1 = very likely or very unlikely to receive the vaccine, 0 = unsure, somewhat likely, somewhat unlikely and not interested within the next two months). The independent variables included the model are gender, age, level of education, and use of social media as primary source of vaccine information. The model was tested with the statistical package STATA v17. We refer the reader to two previous studies based on the same sample for an in-depth analysis of predictors of vaccine acceptance [19,20].

## 3. Results

### 3.1. Sample Characteristics

We gathered responses from 2650 subjects, of which 1591 (60%) expressed some level of hesitancy about receiving the COVID-19 vaccine. The analysis presented in this paper focuses on this group of hesitant individuals. Table 1 shows the sample characteristics (i.e., job categories), and distribution of respondents by vaccine acceptance. In terms of sample characteristics, the majority of respondents were male (55%), in the age group 25–44 (66%), white non-Hispanic (66%), had a college or higher level of education (56%) and were either healthcare workers (including EMS), pharmacists, working in a nursing home or in public health (68%). The rest (32%) were working in other essential services such as transportation, vaccine manufacturing, etc. When asked about sources of information about the vaccine, 69% reported to have received most of their information from traditional media compared to social media. When asked if they will receive the vaccine (vaccine acceptance) in the next two months (if offered to them): 188 (12%) said they would not take it within such timeframe, 339 (21%) were very unlikely to take it, 153 (10%) were somewhat unlikely, 388 (24%) were not sure and 523 (33%) were somewhat likely. 

### 3.2. Top Informational Needs

**Figure 1:**
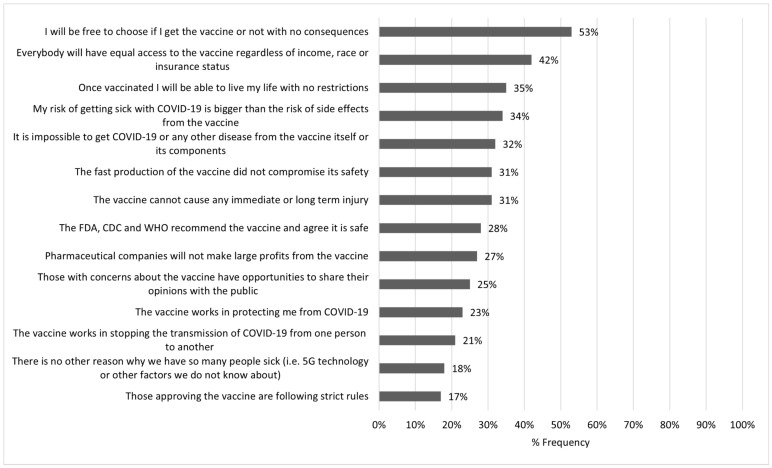
What would be important for you to know to make you more likely to take the COVID-19 vaccine?

As shown in Figure 1, which reports how respondents answered the question “What would be important for you to know to make you more likely to take the COVID-19 vaccine?”, concerns about freedom of choice (*n* = 836, 53%), equal access to the vaccine (*n* = 669, 42%) and being able to live a life with no restrictions once vaccinated (*n* = 559, 35%) were the three most frequently reported issues about which respondents wanted to receive more information (vaccine informational needs).

### 3.3. Informational Needs on the COVID-19 Vaccine Safety and Effectivenes

Table 2 displays the differences in informational needs about the vaccine in terms of its safety and effectiveness. Informational needs are displayed by vaccine acceptance status, sociodemographic characteristics, and the respondent’s use of social media as the main source of vaccine information. 

#### 3.3.1. Informational Needs on Safety and Effectiveness by Vaccine Acceptance

Needs for additional information on the safety and effectiveness of the vaccine were associated with vaccine acceptance (chi-squared, *p*-value < 0.05).

More specifically, respondents reporting to be somewhat likely to take the vaccine manifested interest in knowing more about the protection conferred by the vaccine (*n* = 145; 27.7%) and personal risks and benefits (*n* = 206; 39.4%) compared to those being unlikely to take it (*n* = 58; 17.1% and *n* = 75; 22.1%, respectively) (squared Pearson residuals > 3.84). Respondents saying that they would not take the vaccine within two months but potentially later, expressed the need of reassurance that the vaccine does not cause immediate or long-term injuries (*n* = 74; 39.4%) compared to other groups (squared Pearson residual = 4.63). Those somewhat unlikely to get it were less interested compared to other groups in obtaining information on how the vaccine could or not prevent transmission from one person to another (*n* = 17; 11.1%) (squared Pearson residual = 6.77). 

#### 3.3.2. Informational Needs on Safety and Effectiveness by Sociodemographics

When we analyzed differences in informational needs across sociodemographic groups, we noted differences by gender about concerns the vaccine could cause injuries and about policies assuring equal access to the vaccine (chi-squared, *p*-value = 0.02 and 0.04, respectively). Males had a greater interest in knowing the vaccine does not cause injuries than females (33.0% vs. 27.7%) while females expressed greater interest in being assured that different segments of the population had equal access to the vaccine (44.8% vs. 39.7%).

#### 3.3.3. Informational Needs on Safety and Effectiveness by Use of Social Media

Interestingly, respondents that received most of their information about the vaccine from traditional media or a mix of traditional and social media were more likely, compared to those who obtained most of their information from social media only, to report higher informational needs. More specifically, they wanted to know more about the risks of the fast production of the vaccine (37.9% vs. 29.3%), and the risks and benefits of being vaccinated compared to catching the disease (40.1% vs. 31.7%), the protection conferred by the vaccine (27.6% vs. 21.0%), the vaccine being able to stop the transmission of the disease (25.9% vs. 18.4%) and assurance that the vaccine would not cause COVID-19 (36.4% vs. 29.9%) (all chi-squared *p*-values < 0.05).

### 3.4. Informational Needs on the COVID-19 Vaccine Policies

Table 3 shows differences in terms of additional informational needs related to the vaccine distribution policies by vaccine acceptance, sociodemographic characteristics, and use of social media as main source of vaccine information. 

#### 3.4.1. Informational Needs on Vaccine Policies by Vaccine Acceptance

The need for additional information was associated with vaccine acceptance (chi-squared, *p*-value < 0.05) for five out of the seven vaccine policies topics explored. More specifically, respondents being very unlikely to take the vaccine compared to other groups were less likely to need additional assurance from the FDA, CDC and WHO about the vaccine safety (*n* = 64, 18.9%, squared Pearson residual = 9.90) and less likely to need additional information about how vaccine policies could impact their personal freedom (*n* = 94, 27.7%, squared Pearson residual = 5.29) or about the equal distribution of the vaccine (*n* = 103; 30.4%, squared Pearson residual = 10.97). Additionally, those somewhat unlikely to get it showed less informational needs about the equal distribution of the vaccine (*n* = 48; 31.4%, squared Pearson residual = 4.15). On the contrary, those somewhat likely to get vaccinated did manifest the need for additional information about equal access in the distribution of the vaccine (*n* = 267; 51.1%, squared Pearson residual = 10.08). Respondents saying that they will not take the vaccine within two months but potentially later, expressed a greater need for additional information about whether those approving the vaccine were following strict rules (*n* = 53, 28.2%, squared Pearson residual = 12.55). In addition, those somewhat likely to get it could be convinced by receiving more information on agreement on the vaccine safety by the FDA, CDC and WHO (*n* = 177; 33.8%, squared Pearson residual = 6.60) and about being able to live a life with no restrictions once vaccinated (*n* = 214; 40.9%, squared Pearson residual = 4.98) while they were less likely to need reassurance that those approving the vaccine were following strict rules (*n* = 70, 13.4%, squared Pearson residual = 4.87). 

* *p* < 0.05 for Pearson chi-squared test of independence. ^†^ *p* < 0.05 for Cochran–Armitage Trend Test. ^▲/▼^ denotes that this category exhibits a much higher/lower number of observed counts compared to the other categories in the same column based on the squared Pearson residuals for this cell being greater than 3.84 when the overall chi-squared test is significant.

#### 3.4.2. Informational Needs on Vaccine Policies by Sociodemographics

The needs of information about the impact of policies on issues related to freedom of choice (i.e., being free to decide if getting the vaccine or not) and freedom of speech—expressed as the need to know that those with concerns about the vaccine would be able to share their opinions—differed by level of education (chi-squared *p*-value = 0.03 and 0.004, respectively). For freedom of speech, those with some college education had higher informational needs (*n* = 103, 31.6%, squared Pearson residuals = 4.74) compared to others.

#### 3.4.3. Informational Needs on Vaccine Policies by Use of Social Media

Individuals who did not obtain most of their information about the COVID-19 vaccine from social media, compared to those who did, wanted to be reassured that international organizations agreed about the safety of the vaccine (i.e., FDA, CDC and WHO) (33.7% vs. 25.7%), that they could live a life with no restrictions once vaccinated (41.1% vs. 34.6%), that pharmaceutical companies will not make large profits from the vaccine (30.3% vs. 24.1%) and that everyone would have equal access to the vaccine (53.5% vs. 52.0%).

#### 3.4.4. Informational Needs Found to Be Positively Associated with Vaccine Acceptance in the Univariate Analysis

In Table 4 we provide a summary of the informational needs with a positive association with vaccine acceptance.

### 3.5. Logistic Regression Model

Table 5 presents the results of the multivariable model performed to assess the association between sociodemographic variables, informational needs and strength of opinions about taking the vaccine (vaccine certainty). Overall, 53% of respondents had strong opinions for either taking or not taking the vaccine (vaccine certainty), while 47% were uncertain. The goodness of fit of the model results were: Hosmer–Lemeshow (*p* = 0.46) and Pulstenis–Robinson (*p* = 0.23). In the multivariable model the only variables associated with vaccine certainty were informational needs and use of social media as main source of COVID-19 vaccine information. The variable informational needs was created as a dichotomous variable (1 = the respondent had informational needs in at least one of the seven topic areas associated with vaccine acceptance described in Table 4, 0 = the respondent had no informational needs in any of the seven topic areas). Results show that individuals reporting to have received most of their information on the vaccine from social media were 1.2 times more likely to have an opinion about getting or not the vaccine (very likely to get it and very likely not to get it) compared to individuals using traditional media or a mix of traditional and social media (OR = 1.2, 95% C.I. 0.9–1.5). Individuals with informational needs in one or more of the seven topic areas identified had 98% decreased odds of being certain about receiving or not receiving the vaccine (OR = 0.02, 95% C.I. = 0.01 = 0.03).

## 4. Discussion

The ability to develop, coordinate, and disseminate information, alerts, warnings, and notifications to the public and incident management personnel (Capability 4: Emergency Public Information and Warning) is a key CDC preparedness capability that public health agencies across the country will need to continue to implement in the months and years ahead to support the COVID-19 vaccination campaign [21]. Even though our survey was conducted prior to the COVID-19 initial vaccine rollout in December 2020, at a time when demand for the vaccine was high and supply low, our data are consistent with more recent polls stating that approximately 11–20% of the U.S. population is not interested in getting the vaccine or is somewhat hesitant. This study has important practical implications for those developing vaccine communication strategies because it aimed at understanding what types of information the hesitant individuals would need to make them more likely to accept the vaccine. Our results may also be informative to any future vaccination campaign when a new vaccine is introduced in the market, including future new COVID-19 vaccines and the use of booster shots. 

It is noteworthy that the top informational needs identified in our sample were not related to pharmaceutical or medical issues, such as the safety or effectiveness of the vaccine, but rather to the impact of vaccine policies on everyday life. Further, our survey showed that concerns related to freedom of health choice, freedom of speech and general freedom were the most important among the hesitant individuals, even at a time when data on the safety and effectiveness of the vaccine were limited compared to today. This may help to explain why the proportion of people who were hesitant in December 2020 remains relatively consistent at the time of this writing (December 2021) [2] If policies related to vaccine mandates are to persist and be further implemented, risk communicators may want to emphasize the freedoms of activity that vaccination status provides to individuals. This focus on a “freer” lifestyle postvaccination is likely to be appealing to the somewhat hesitant population. On the contrary, those who are very hesitant and very unlikely to receive the vaccine do not seem to be interested in receiving additional information either about the vaccine as a pharmaceutical product or about vaccine-related policies. It is worth considering that, in previous studies, demographic characteristics and partisanship have been associated with acceptance of vaccine mandates. Polls conducted prior to the approval of the vaccines, as well as more recent surveys, indicate that some racial–ethnic groups are less likely to accept a potential COVID-19 vaccine [22,23,24,25]. This is particularly concerning, considering that Black individuals shoulder a disproportionate burden of many chronic conditions, placing them at a higher risk for complications from COVID-19 and are also highly represented in essential jobs. This suggests that, in some states or localities, COVID-19 vaccine mandates—particularly for adults—may be ineffective in increasing coverage and may worsen the political divide.

Public health communication efforts aimed at encouraging COVID-19 vaccinations may be more effective when reassuring the public that freedom of health choice, speech and movement will be safeguarded. This strategy may convince those whose acceptance is based on ideological concerns, so that they refocus on the medical and clinical benefits of getting vaccinated. The benefits need to be presented transparently, acknowledging the unknown of a product for which data are limited to a short period of observation, and the risks of experiencing side effects from the vaccine compared to what is known at a given point in time on the benefits in being protected. Those that are willing (somewhat likely to receive the vaccine) are interested in knowing how protective the vaccine is and if it is worth receiving it based on their personal risk. This is important to know because it means that communication efforts focused on narratives emphasizing the risk of catching COVID-19 may be more effective when focusing on those most at risk of severe consequences of the disease compared to others. In that respect, it is also important to communicate in a transparent manner that some policy decisions are made knowing that the benefit to many may be low and that policies are driven by the fact that the ability to predict the epidemiology of the virus and its variants is very limited. 

Regarding the vaccine safety, it is worth noting that the approval process by regulatory agencies was also important to approximately one-third (28%) of our respondents, who wanted to receive reassurance that the FDA and other international organizations approved the vaccines. As such, consistency in the messages delivered by major international organizations is important to support the vaccination campaign.

Interestingly, in our sample, people who obtained most of their information about the vaccine from social media were less likely to report that they needed additional information, regardless of their willingness to be vaccinated. This is consistent with prior research that has found that information on social media is reinforcing and targeted. Individuals using social media are more likely to see similar content to their existing views and searches due to social media algorithms reinforcing their beliefs and reducing interest in other opinions. We interpret this result as a sign of information overload which is a byproduct of a saturated social media environment. Information overload has been studied for its implications on the ability to process information and act on the information received [26,27,28].

We believe the data provided by our study are relevant to the current discourse on vaccine acceptance because early adopters and rejectors of immunizations—especially those belonging to healthcare professional groups—can have a strong influence on the likelihood that others will accept the vaccine. While we wait for the combination of cross-reactive immunity and natural reinfections to take us toward an endemic version of SARS-CoV-2, we are still in a critical response phase, and will be for a few months ahead, where we need to succeed in protecting the most vulnerable individuals, and need to avoid further societal restrictions to mobility with inevitable and negative economic consequences related to closure of businesses and activities.

This study illustrates that vaccine acceptance is a complex construct that reflects not just the acceptance toward a pharmaceutical product but also toward the policies that are developed and used for its distribution. This is clearly a scenario in which ideology influences medical choices with implications for vaccine policy. Mandating vaccination may appear to be an easier strategy to increase vaccine uptake compared to developing better communication efforts. However, it is a strategy with the potential to backfire and further polarize society.

### 4.1. Limitations

This study has several limitations. First, our sample is not representative of all essential workers in the USA. However, a representative sample of essential workers would be very difficult to obtain given the multitude of job categories included in the list of essential workers and the lack of data on how many people work within each category in the nation. Second, while it is true that the percentage of hesitant individuals we found back in December 2020, when the survey was implemented, is very similar to what was found in more recent polls (October 2021), the lack of longitudinal data does not allow us to study changes in the willingness to be vaccinated, therefore we do not know if those who were hesitant in December are the same people who are hesitant now, or if the reasons for acceptance that we explored are reflective of current opinions. In terms of measurement of exposure to social media versus traditional media, this study has also the limitation of not being able to differentiate those that, through the use of social media, access journal articles. In addition, our study certainly suggests that vaccine acceptance is a complex construct and cannot be measured with a single exact measurement or a simple regression analysis. The main limitation of our regression model is the fact that the variable describing informational needs is likely to be part of the construct of the dependent variable describing vaccine opinions (vaccine certainty). Future studies should explore the use of structural equation modeling and path analysis to assess how specific beliefs and informational needs are directly or indirectly impacting acceptance. Finally, our results might not be comparable to other national polls or surveys because of potential differences in the survey methods, sample populations and questions related to vaccination intent.

### 4.2. Recommendations for Practice

It is important to acknowledge that the need to mandate vaccinations is a direct consequence of the failure to convince and reassure the public about the importance of getting vaccinated. Such failure belongs to the response area of risk-communication. Since the early days of the vaccination campaign, a multitude of communication and outreach strategies have been implemented to increase vaccination rates, including vaccination fairs, continuous advertisement on TV, social media, and radio stations, reminders sent to patients and clients by healthcare providers, as well as lotteries and other types of incentives which some have also argued to be unethical [28]. In this section, we extrapolate some lessons learned from the data we presented, based on our interpretation of the results and considerations for public health practice. 

The first recommendation derived from this manuscript is for public health officials and their spokespersons to maintain transparency during all communication efforts related to the COVID-19 vaccine, by acknowledging the risks and benefits of the different types of vaccines, including short-term side effects and rare but possible complications, and to identify the specific topic areas people need additional information on, avoiding over-reassurance on general safety and effectiveness but rather providing information based on specific informational needs. The second recommendation is for businesses’ human resources personnel and politicians who will need to develop strategies to mitigate the long-term psychological consequences of the mandates. While mandates have certainly increased vaccination rates in the short term, due to people’s fear to lose their job, it is very likely they may have a long-term negative impact on the trust and relationship between workers, citizens and the institutions and organizations they interact with. The third recommendation is for those who develop the content of the communication campaigns, to focus such content on the practical positive implications of vaccination efforts (i.e., reduced disruption of work-related activities, social impact) rather than on the ideology behind the decisions being made (i.e., individual versus public benefit), as the mixing of ideology and science is likely to cause confusion and lack of understanding of the benefits of mass vaccination efforts. The fourth recommendation is to develop strategies in the use of social media to disseminate the message across a variety of groups, as individuals with the same opinions are likely to see information only limited to their group’s echo chamber.

## 5. Conclusions

The ability to convey public health information is a key preparedness capability that public health agencies across the country will need to continue to implement in the months ahead to support the COVID-19 vaccination campaign. Vaccine acceptance is a complex construct that reflects not just the acceptance toward a pharmaceutical product but also toward the policies that are developed and used for its distribution. It is noteworthy that the top informational needs identified in our sample were not related to the safety or effectiveness of the vaccine as a pharmaceutical product, but, rather, to vaccine policies that can impact freedom of speech, freedom of health choices and general freedom. Mandating vaccination may appear to be an easy solution to increase vaccine uptake. However, it is a strategy with the potential to backfire and further polarize society because it brings up ideological concerns. Ideology distracts the public from the most important and scientific components of the communication strategy which should focus on the risks and benefits of the vaccine as a pharmaceutical product.

## Figures and Tables

**Table 1:** Sample characteristics and vaccine acceptance.

Demographics/Characteristics	*n* (%)
Age	
18–24	212 (13.3)
25–34	518 (32.6)
35–44	539 (33.9)
45–54	199 (12.5)
55+	123 (7.7)
**Gender** **(*N* = 1581)**	
Male	874 (55.3)
Female	707 (44.7)
**Race/ethnicity**	
White, Non-H	1048 (65.9)
Black, Non-H	242 (15.2)
Hispanic	119 (7.5)
Non-H Asian	73 (4.6)
Multi/Other	109 (6.9)
**Education** **(*N* = 1584)**	
HS/less	375 (23.7)
Some college	326 (20.6)
Bachelor’s degree	363 (22.9)
Postgraduate degree	520 (32.8)
**Vaccine Acceptance**	
I would not take it within 2 months	188 (11.8)
Very unlikely	339 (21.3)
Somewhat unlikely	153 (9.6)
I am not sure	388 (24.4)
Somewhat likely	523 (32.9)
Get most information about COVID-19 vaccine from social media? * **(*N* = 1334)**	
No	925 (69.3)
Yes	409 (30.7)
**Job category (multiple choice question)**	
Hospital and emergency department workers	624 (23.5)
Nursing home, long-term care, and home healthcare workers	413 (15.6)
Public health workers	284 (10.7)
Grocery store workers	283 (10.7)
Teachers and school staff	251 (9.5)
Food processing workers	222 (8.4)
Emergency medical services workers	186 (7.0)
Other healthcare workers	170 (6.4)
Volunteer (i.e., CERT, MRC, Red Cross, etc.)	168 (6.3)
Private transportation workers	156 (5.9)
Sanitation workers	131 (4.9)
Vaccine manufacturing workers	121 (4.6)
Postal and shipping workers	120 (4.5)
Pharmacy workers	117 (4.4)
Correctional facilities workers	116 (4.4)
Police or firefighters	116 (4.4)
Vaccine distribution workers	95 (3.6)
Other first responders	93 (3.5)
Public transportation workers	90 (3.4)

* We created a variable to characterize the main source of information an individual selected about the COVID-19 vaccine: obtaining most information from only traditional media channels (TV, newspaper, or radio), obtaining most information from only social media channels (Facebook, Instagram, YouTube, Twitter, or Tik-Tok); and obtaining most information from both social and traditional media channels. For the purposes of this manuscript, we dichotomized those who obtained most of their information from social media versus those that obtained it from traditional media or both social media and traditional media.

**Table 2:** Differences in informational needs related to the COVID-19 vaccine safety and effectiveness by vaccine acceptance, sociodemographics, and use of social media.

What Would Be Important for You to Know to Make You More Likely to Take the COVID-19 Vaccine? (*N* = 1591)	
Vaccine Acceptance	My risk of getting sick with COVID-19 is bigger than the risk of side effects from the vaccine. *n* (%) *^,†^
I would not take it within 2 months	56 (29.8)
Very unlikely	75 (22.1) ^▼^
Somewhat unlikely	56 (36.6)
I am not sure	147 (37.9)
Somewhat likely	206 (39.4) ^▲^
**Vaccine Acceptance**	The vaccine cannot cause any immediate or long-term injury. *n* (%) *^,†^
I would not take it within 2 months	74 (39.4) ^▼^
Very unlikely	116 (34.2)
Somewhat unlikely	36 (23.5)
I am not sure	126 (32.5)
Somewhat likely	136 (26.0)
**Vaccine Acceptance**	**There is no other reason why we have so many people sick (i.e., 5G technology or other logy or other factors we do not know about). *n* (%) ^†^**
I would not take it within 2 months	32 (17.0)
Very unlikely	49 (14.5)
Somewhat unlikely	34 (22.2)
I am not sure	69 (17.8)
Somewhat likely	95 (18.2)
**Vaccine Acceptance**	The vaccine works in protecting me from COVID-19. *n* (%) *^,†^
I would not take it within 2 months	44 (23.4)
Very unlikely	58 (17.1) ^▼^
Somewhat unlikely	35 (22.9)
I am not sure	84 (21.6)
Somewhat likely	145 (27.7) ^▲^
**Vaccine Acceptance**	The vaccine works in stopping the transmission of COVID-19 from one person to another. *n* (%) *
I would not take it within 2 months	42 (22.3)
Very unlikely	69 (20.4)
Somewhat unlikely	17 (11.1) ^▼^
I am not sure	83 (21.4)
Somewhat likely	118 (22.6)
**Vaccine Acceptance**	**It is impossible to get COVID-19 or any other disease from the vaccine itself or its components. *n* (%) ^†^**
I would not take it within 2 months	57 (30.3)
Very unlikely	97 (28.6)
Somewhat unlikely	42 (27.5)
I am not sure	120 (30.9)
Somewhat likely	193 (36.9)
**Gender (*N* = 1581)**	The vaccine cannot cause any immediate or long-term injury. *n* (%) *
Male	288 (33.0)
Female	196 (27.7)
**Education (*N* = 1584)**	**The vaccine works in protecting me from COVID-19. *n* (%) ^†^**
HS/less	95 (25.3)
Some college	78 (23.9)
Bachelor’s degree	92 (25.3)
Postgraduate degree	100 (19.2)
**Get most information about COVID-19 vaccine from social media? (*N* = 1334)**	The fast production of the vaccine did not compromise its safety. *n* (%) *
No	155 (37.9) ^▲^
Yes	271 (29.3)
**Get most information about COVID-19 vaccine from social media? (*N* = 1334)**	My risk of getting sick with COVID-19 is bigger than the risk of side effects from the vaccine. *n* (%) *
No	164 (40.1) ^▲^
Yes	293 (31.7)
**Get most information about COVID-19 vaccine from social media? (*N* = 1334)**	The vaccine works in protecting me from COVID-19. *n* (%) *
No	113 (27.6)
Yes	194 (21.0)
**Get most information about COVID-19 vaccine from social media? (*N* = 1334)**	The vaccine works in stopping the transmission of COVID-19 from one person to another. *n* (%) *
No	106 (25.9) ^▲^
Yes	170 (18.4)
**Get most information about COVID-19 vaccine from social media? (*N* = 1334)**	It is impossible to get COVID-19 or any other disease from the vaccine itself or its components. *n* (%) *
No	149 (36.4)
Yes	277 (29.9)

* *p* < 0.05 for Pearson chi-squared test of independence. ^†^ *p* < 0.05 for Cochran–Armitage Trend Test. ^▲/▼^ denotes that this category exhibits a much higher/lower number of observed counts based on the squared Pearson residuals for this cell being greater than 3.84 when the overall chi-squared test is significant.

**Table 3:** COVID-19 vaccine policies: differences in informational needs about the impact of vaccine policies on everyday life by sociodemographics, use of social media and vaccine acceptance.

What Would Be Important for You to Know to Make You More Likely to Take the COVID-19 Vaccine? (*N* = 1591).							
Demographics/Characteristics	Those Approving the Vaccine Are Following Strict Rules	The FDA, CDC and WHO Recommend the Vaccine and Agree It Is Safe	Once Vaccinated I Will Be Able To Live My Life With No Restrict-Ions (General Freedom)	Those With Concerns About the Vaccine Have Opportunities To Share Their Opinions With the Public (Freedom of Speech)	Pharmaceutical Companies Will Not Make Large Profits From the Vaccine	Everybody Will Have Equal Access to the Vaccine Regardless of Income, Race or Insurance Status (Equal Access)	I Will Be Free To Choose if I Receive the Vaccine or Not With No Consequences (Freedom of Health Choices)
Vaccine Acceptance *n* (%) *^,†^		*n* (%) *^,†^	*n* (%) *^,†^	*n* (%)	*n* (%)	*n* (%) *^,†^	*n* (%) *^,†^
I would not take it within 2 months	53 (28.2) ^▲^	51 (27.1)	62 (33.0)	55 (29.3)	54 (28.7)	83 (44.1)	101 (53.7)
Very unlikely	64 (18.9)	64 (18.9) ^▼^	94 (27.7) ^▼^	96 (28.3)	81 (23.9)	103 (30.4) ^▼^	197 (58.1)
Somewhat unlikely	22 (14.4)	30 (19.6)	49 (32.0)	31 (20.3)	40 (26.1)	48 (31.4) ^▼^	85 (55.6)
I am not sure	68 (17.5)	122 (31.4)	140 (36.1)	103 (26.5)	90 (23.2)	168 (43.3)	204 (52.6)
Somewhat likely	70 (13.4) ^▼^	177 (33.8) ^▲^	214 (40.9) ^▲^	120 (22.9)	158 (30.2)	267 (51.1) ^▲^	249 (47.6)
Education (*N* = 1584)		^†^		*^,†^			^*^
HS/less	62 (16.5)	119 (31.7)	134 (35.7)	106 (28.3)	95 (25.3)	162 (43.2)	202 (53.9)
Some college	59 (18.1)	98 (30.1)	104 (31.9)	103 (31.6) ^▲^	93 (28.5)	151 (46.3)	156 (47.9)
Bachelor’s degree	59 (16.3)	94 (25.9)	135 (37.2)	80 (22.0)	89 (24.5)	155 (42.7)	212 (58.4)
Postgraduate degree	96 (18.5)	132 (25.4)	184 (35.4)	115 (22.1)	145 (27.9)	200 (38.5)	263 (50.6)
Get most information about COVID-19 vaccine from social media? (*N* = 1334)							
No	68 (16.6)	138 (33.7) ^▲^	168 (41.1)	112 (27.4)	124 (30.3)	219 (53.5) ^▲^	222 (54.3)
Yes	157 (17.0)	238 (25.7)	320 (34.6)	224 (24.2)	223 (24.1)	344 (37.2) ^▼^	481 (52.0)

* *p* < 0.05 for Pearson chi-squared test of independence. ^†^ *p* < 0.05 for Cochran–Armitage Trend Test. ^▲/▼^ denotes that this category exhibits a much higher/lower number of observed counts compared to the other categories in the same column based on the squared Pearson residuals for this cell being greater than 3.84 when the overall chi-squared test is significant.

**Table 4:** Positive associations between informational needs and likelihood of taking the vaccine.

How Likely Are You to Take a COVID-19 Vaccine If Offered to You at No Cost within Two Months (Very Likely, Somewhat Likely, I Would Not Take It within Two Months but Consider It Later, Not Sure, Somewhat Unlikely, Very Unlikely)	What Would Be Important for You to Know to Make You More Likely to Take the COVID-19 Vaccine?
Respondents reporting somewhat likely	My risk of getting COVID-19 is bigger than the risk of side effects from the vaccine. The vaccine cannot cause any immediate or long-term injury. The vaccine works in protecting me from COVID-19. The FDA, CDC and WHO recommend the vaccine and agree it is safe. Once vaccinated I will be able to live my life with no restrictions (general freedom). Everybody will have equal access to the vaccine regardless of income, race or insurance status.
I would not take it within two months but consider it later	The vaccine cannot cause any immediate or long-term injury. Those approving the vaccine are following strict rules.

**Table 5:** Multivariable Model—Association between sociodemographics, use of social media and vaccine certainty.

Variable	OR	95% C.I.
Age	0.97	0.88–1.08
Gender (Female versus Male)	0.99	0.79–1.26
White non-Hispanic versus other races	0.89	0.70–1.15
Education		
Less than high school	-	-
High school/GED	1.15	0.59–2.27
Some college	1.07	0.54–2.11
Bachelor’s degree	1.06	0.54–2.08
Postgraduate degree	1.33	0.69–2.60
Social media as main source of COVID-19 vaccine information versus traditional media/mixed media	1.18	0.91–1.52
Informational needs (need of additional information in at least one of the seven topics selected versus no need of additional information)	0.02	0.01–0.03

## Supplementary Materials

A copy of the questionnaire is available online at: https://github.com/esavoia123/Vaccine-hesitancy-data-Dec-2020-USA.
